# SAVI: a statistical algorithm for variant frequency identification

**DOI:** 10.1186/1752-0509-7-S2-S2

**Published:** 2013-10-14

**Authors:** Vladimir Trifonov, Laura Pasqualucci, Enrico Tiacci, Brunangelo Falini, Raul Rabadan

**Affiliations:** 1 Department of Biomedical Informatics, Columbia University, New York, NY, 10032, USA; 2 Department of Systems Biology, Columbia University, New York, NY, 10032, USA; 3 Center for Computational Biology and Bioinformatics, Columbia University, New York, NY, 10032, USA; 4 Institute for Cancer Genetics and Herbert Irving Comprehensive Cancer Center, Columbia University College of Physicians and Surgeons, New York, NY, USA; 5 Department of Pathology and Cell Biology, Columbia University, New York, NY, 10032, USA; 6 Institute of Hematology, University of Perugia, Perugia, Italy

## Abstract

**Background:**

Many problems in biomedical research can be posed as a comparison between related samples (healthy vs. disease, subtypes of the same disease, longitudinal data representing the progression of a disease, etc). In the cases in which the distinction has a genetic or epigenetic basis, next-generation sequencing technologies have become a major tool for obtaining the difference between the samples. A commonly occurring application is the identification of somatic mutations occurring in tumor tissue samples driving a single cell to expand clonally. In this case, the progression of the disease can be traced through the trajectory of the frequency of the oncogenic alleles. Thus obtaining precise estimates of the frequency of abnormal alleles at various stages of the disease is paramount to understanding the processes driving it. Although the procedure is conceptually simple, technical difficulties arise due to inhomogeneous samples, existence of competing subclonal populations, and systematic and non-systematic errors introduced by the sequencing technologies.

**Results:**

We present a method, Statistical Algorithm for Variant Frequency Identification (SAVI), to estimate the frequency of alleles in a set of samples. The method employs Bayesian analysis and uses an iterative procedure to derive empirical priors. The approach allows for the comparison of allele frequencies across several samples, e.g. normal/tumor pairs and more complex experimental designs comparing multiple samples in tumor progression, as well as analyzing sequencing data from RNA sequencing experiments.

**Conclusions:**

Analyzing sequencing data through estimating allele frequencies using empirical Bayes methods is a powerful complement to the ever-increasing throughput of the sequencing technologies.

## Background

High-throughput sequencing (HTS) has become one of the most important tools in the arsenal of biomedical researchers in their quest for understanding the foundations of diseases with a strong genetic component like cancer [1]. Sequencing technologies have significantly increased the amount of data produced by a single run of an instrument with their modern high-throughput versions producing more than 1Tb in a single run. Thus, in the case of the human genome, the scale of the sequencing experiments has been pushed into the realm of whole-exome [2,3], whole transcriptome [4,5], and even whole-genome studies [6,7]. The sheer volume of this data combined with the inherently random aspect of the sequencing process conspire to introduce uncertainties in the output of the experiments. Furthermore, the intricate chemistry of the novel sequencing instruments often results in systematic errors, which can make artifacts indistinguishable from actual genetic lesions. Although detecting such sources of non-random errors is an important part of analyzing the data produced by sequencing experiments, the purpose of this paper is to develop a framework for the correction of the random component in the noise introduced by the sequencers. Furthermore, our goal is not to establish the biochemical sources of those errors, but to develop a statistical approach applicable in diverse scenarios in which the assumption about the random nature of the source of errors holds.

A major application of HTS is to compare related samples, e.g. healthy vs. disease or various stages in the progression of a disease, based on their genetic makeup. Many such examples are provided in cancer research, where identifying the genetic or epigenetic lesions that contribute to the oncongenic process is an important step towards better diagnosis, evaluation of prognosis, and treatment of the disease. Thus HTS has become a powerful unbiased approach to delineating the landscape of genetic alterations in different tumor types [8,9]. An often-applied technique involves identifying aberrant somatic alleles fixed in a population of cancer cells, which are absent from a control sample. The wisdom behind this technique is based soundly on the observation that the drivers of oncogenic processes are fixed by the clonal expansion [10]. From a more general perspective, and in addition to making a digital (binary) present/absent call, one could strive for identifying the frequency of alleles in a given population of cells. The importance of this analysis to cancer research is immense since low frequency alleles can have a major contribution to the disease in later stages [11], e.g. by conferring resistance to treatment. Furthermore, detecting low frequency alleles at an early stage of the disease, and even before the disease has manifested itself, can be crucial for its prognosis.

**Figure 1:**
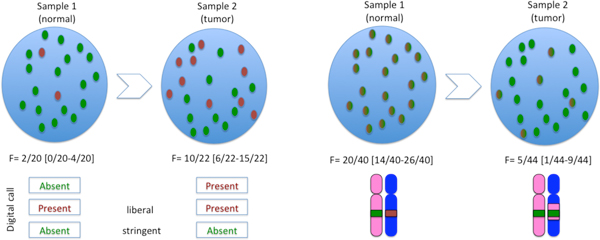
**High-throughput sequencing data provides information about the frequency of an allele in a population of cells**. The numbers in parentheses are the 5% confidence interval. Depending on parameters, discretization might distort our perception of the data (left). Alleles can be gained by point mutation (left) or lost by LOH, e.g. copy-neutral LOH (right), in the evolution of the population.

The comparison of two temporally ordered samples with respect to the frequency of an allele could indicate either a gain or a loss of that allele. Both of these alternatives could be the cause (driver) or a consequence (passenger) of the biological process driving the phenotype. For example, a point mutation could inactivate the normal function of a tumor suppressor gene and lead to the formation of cells with a cancer phenotype (Figure 1, left). Alternatively, the abnormal phenotype could be the result of a loss of heterozygosity (Figure 1, right). Although, distinguishing driver from passenger alterations is an important part of the functional analysis of clonally expanding populations of cells, detecting such events regardless of their direction and relevance to the phenotype is the first step towards such analysis.

Besides the clinical importance of diagnosing and treating cancer, having a detailed picture of the alleles present at different stages of the evolution of a population of cells is an important step towards understanding the intricate biological processes underlying its existence. Several approaches have been used to identify the genetic alterations differentiating a set of related samples. Some methods [12,4] involve ad hoc thresholds on the number of reads reporting a variant allele, their quality and/or the total number of reads covering the position of the variant allele to produce a binary present/absent call for that variant. A comparison between different samples is then based on differences in those calls (see Figure 1). Arbitrary thresholds on the number of reads reporting a variant introduce biases due to the uneven distribution of depths along the genome. Such methods can miss variants which have somewhat lower quality but high depth or lower depth, but high quality. Furthermore, decisions based on a discretized view of the data depend strongly on the level of discretization and other parameters of the discretization process. This could be a problem if the samples being compared are not perfectly homogeneous, e.g. by contamination or the natural presence of tumor cells in the control sample. Other methods [13,14] assume allele distributions particular to a homogenous population of diploid genomes. Similar to our approach, the algorithm SNVMix [15] uses a Bayesian framework and is able to identify variants in samples with different ploidy and tumor cellularity. The algorithm employs an expectation-maximization for constructing a prior.

In this paper we present the statistical Algorithm for Variant frequency Identification (SAVI) developed in the course of analyzing data from sequencing experiments of cancer samples from nine Hairy Cell Leukemia (HCL) patients and the corresponding paired normal samples. The initial findings are published in [2]. The algorithm is based on constructing Bayesian posteriors distributions on allele frequencies and employs an iterative procedure for constructing an empirical prior from a given dataset. Having posterior distributions lets us obtain a high credibility interval for the frequency of a particular allele as well as estimates on its expected and most likely value. In contrast to other applications of Bayesian analysis to the problem of frequency estimation, our prior is not fixed to belong to a predetermined set that might not represent an accurate description of the data. Our desire was to develop a method which is more empirically grounded and governed by the data itself as much as possible. With the advent of large datasets the significance of Bayesian methods using priors based on empirical observations has increased [16,17]. Thus our goal to tailor the prior to the data was naturally steered towards such considerations. Empirical Bayesian methods have been applied before in the context of gene expression in RNA sequencing experiments [18] and estimates of positive selection in tumors [19]. Although, as outlined above, the techniques developed in this manuscript are quite general, we focus here on two important aspects of its application: detection and genotyping of variant alleles in a population of cells and comparing allele frequencies across different samples.

## Results

The methods described in this paper were developed to analyze the sequencing data from a study on HCL - a lymphoid malignancy in which bone marrow, spleen and liver are infiltrated by leukemic B cells showing abundant cytoplasm with characteristic "hairy" projections. In the study, the exome of samples from peripheral blood leukemic hairy cells and paired normal mononuclear cells from 9 HCL patients was sequenced with Illumina GAIIx and HiSeq2000. Genetic material from the exome of those samples was obtained by enrichment with SureSelect. The mate-pair reads produced by the sequencer were aligned to the hg18/NCBI 36.1 human reference using MAQ. On average around 27M of the 28M exonic positions reported in the NCBI 36.3 CCDS database were covered by reads with an average haploid depth of 55. After filtering, described next, on average 16K exonic positions were determined to contain a variant. Of those, 2,500 were novel (not recorded in dbSNP 130) and 1,900 were in addition non-synonymous. Finally, across all samples a total of 81 of the novel non-synonymous variants were predicted to be somatic. These variants were compared against a list of 121 tested variants 67 of which were confirmed to be somatic. Fifty-six of the confirmed somatic variants were predicted and only 1 of the tested, but not confirmed variants, was predicted.

To analyze the variants produced by the sequencer we applied the Bayesian framework outlined in the Methods section. As discussed there, for every potential variant allele we obtained the number of reads confirming the variant, the average Phred score of the nucleotides containing the variant, and the total number of reads covering its locus. Assuming a binomial likelihood of the variant depth given a particular allele frequency, total depth, and a prior distribution on those frequencies, we obtained a posterior distribution for the allele frequency for every tentative allele using Bayes theorem. In the following discussion, the priors and posteriors have precision 1% unless explicitly stated otherwise.

**Figure 2:**
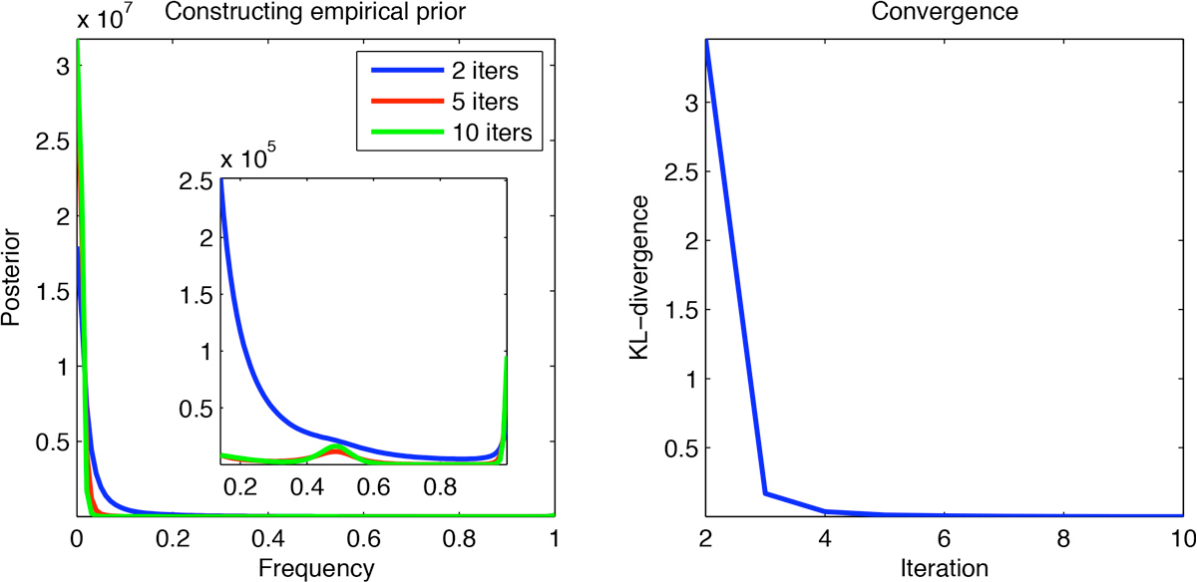
**Iterative procedure for constructing an empirical prior**. Features particular to a diploid genome compared to a haploid reference become pronounced (left). The procedure is converging to a fixed point (right).

To construct a prior empirically we apply an iterative procedure based on the observation that a prior appropriate to a given set of observations should predict the empirically observed distribution of that data as a certain marginal distribution. The iterative procedure derived from this observation has as a fixed point a prior satisfying it. The details of the procedure are given in the Methods section. Figure 2 (left) shows the result of iterations 2, 5, and 10 of the procedure starting with a prior uniform over all frequencies. As can be seen, the priors obtained from the procedure get consecutively better at picking up the salient features of the allele frequency distribution particular to a diploid genome compared to a haploid reference - most positions are homozygous for the reference, a number of positions are heterozygous, and that number is proportional to the homozygous variant positions. Figure 2 (right) contains the Kullback-Leibler divergence between every two consecutive priors produced by the procedure. As can be seen in this plot, in our case the procedure is converging to a fixed point. Analyzing the convergence properties of this procedure in more detail is certainly of high mathematical interest but beyond the scope of this paper.

**Figure 3:**
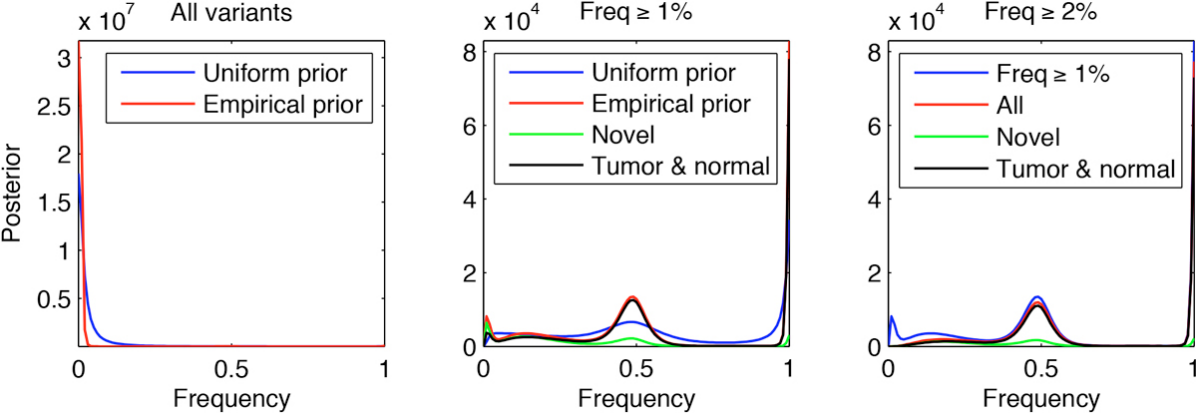
**Aggregated posteriors before filtering (left) and after filtering (center)**. Filtering at 2% frequency derived from locations present in dbSNP removes potential sequencing artifacts (right).

Figure 3 (left) shows the aggregation of the posterior distributions by adding the corresponding posterior probabilities across all exonic locations. The figure contains two plots: one in which the prior was assumed to be uniform (in blue) and one (in red) which was obtained empirically. At the scale of the figure, the aggregated posterior obtained from the empirical prior exhibits a shift towards frequency 0 (non-variant alleles) when compared to the aggregated posterior obtained from the uniform prior. Considering that a posterior obtained from the uniform prior peaks at the observed frequency of the allele, we can say that the empirical prior shifts the posteriors of alleles with low frequency towards the 0 frequency since the empirical prior gives more weight to that frequency. The fact that the 0 frequency gets more weight in the empirical prior as compared to the uniform is expected, since most alleles are homozygous and equal to the reference. Thus the information accumulated from all locations is leveraged in a particular locus as an additional corrective factor. This ability of the empirical Bayesian framework to borrow information from all experiments in the decision for a particular experiment and to shrink the observations towards a common mean is considered one of its strongest theoretical and practical properties [17,20].

### Detection of variants

Given the posterior distribution for the frequency of an allele we obtained the posterior probability that the allele has a non-zero frequency. Our decision was to consider as present variants whose posterior probability of having a non-zero frequency was at least 1-10^-6 ^(see the Methods section for the choice of confidence cutoffs). Figure 3 (center) shows in red the aggregated posterior obtained from variants determined to be present with the criterion that the hypothesis "frequency at least 1%" is credible at the 1-10^-6 ^level and using the empirical prior. The plot also contains (in blue) the aggregated posterior corresponding to the uniform prior. A comparison between the two posteriors shows again the shrinking towards a common mean, this time towards the frequency 50% for heterozygous variants and towards 100% for homozygous variant alleles. Also shown here are the aggregated posteriors for variants present in both the tumor and normal samples (in black) and novel variants not documented in the NCBI dbSNP130 database (in green).

**Figure 4:**
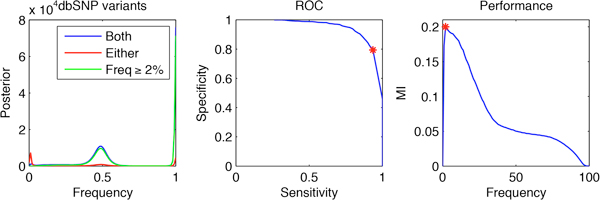
**Establishing frequency cutoff using known SNPs**. SNPs present in both samples are more likely to be genuine (right). ROC curve for a varying allele frequency cutoff (center). Obtaining a frequency cutoff by maximizing mutual information (right).

Next we analyzed the performance of the 1% criterion on positions reported in the dbSNP database. Figure 4 (left) shows the aggregated posterior of the dbSNP locations for which an allele has been determined present with that criterion. In this figure such locations have been split in two disjoint datasets - those present in both the tumor and normal samples and those present in only one of the samples. Our intuition is that that the variants confirmed in both samples are more likely to be genuine germline polymorphisms. An inspection of the figure shows that the posterior distribution of variants at known locations, which are detected in only one sample exhibits, a peak at low frequencies, confirming our intuition that those variants are likely to be sequencing artifacts. To remove such artifacts we considered the option of raising the cutoff frequency in the filtering criterion. More precisely, our strategy was to obtain for every potential variant the highest frequency *f *for which the hypothesis "frequency at least *f" *is credible a posteriori with confidence at least 1-10^-6 ^- i.e. we obtained a one-sided credible interval for that level. Next, for every possible cutoff frequency we obtained the contingency table for the test corresponding to the cutoff on that frequency vs. the known polymorphic locations present with frequency at least 1% with high posterior confidence in both samples. We computed the mutual information corresponding to that contingency table and chose the frequency cutoff which maximizes it. In our case a frequency cutoff of 2% was chosen. Figure 4 (right) shows the result of this analysis and Figure 4 (center) shows the ROC curve for the frequency cutoff parameter, where the chosen cutoff frequency is marked in red. Finally, we revisited the definition of presence of an allele and used a frequency cutoff of 2% at posterior credibility level 1-10^-6^. The result of this filtering is shown in Figure 3 (right). For comparison, in that plot we have also included the result of filtering with frequency cutoff 1%, which was shown in red in Figure 3 (center). From the plot it is evident that the cutoff at 2% removes a small peak and some of the bulk at low frequencies of the posterior distribution, which we hypothesize are due to sequencing artifacts. A possible extension iterates the process with the newly established cut-off until the aggregated posteriors stop changing substantially as measured, for example, by the Kullback-Leibler divergence.

### Detection of somatic variants

**Figure 5:**
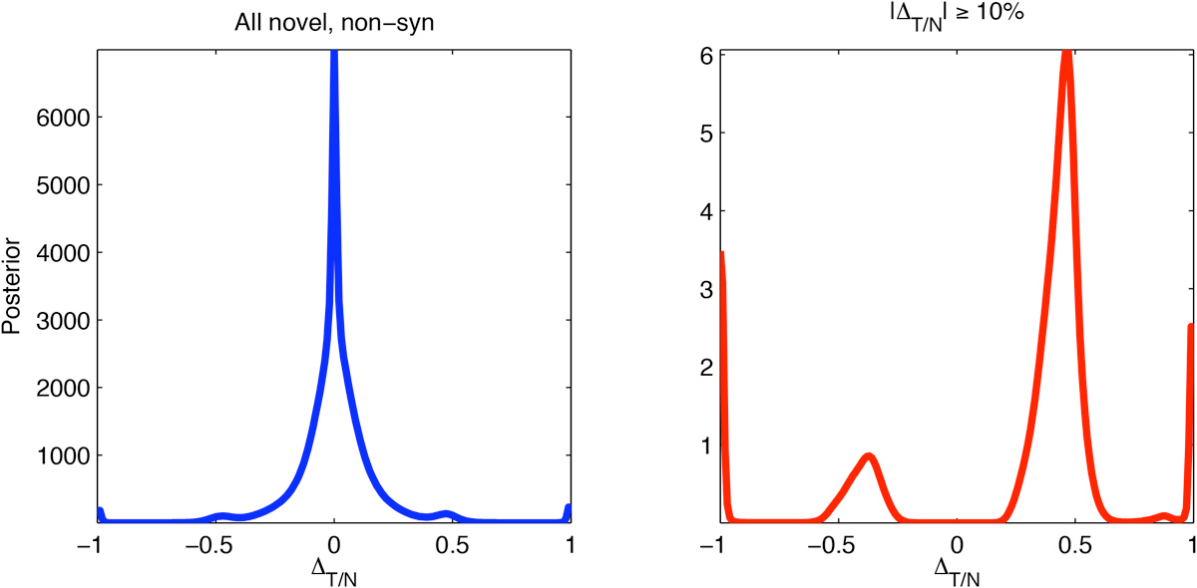
**Aggregate posterior distribution of allele frequency differences for novel, non-synonymous variants before filtering (left) and after filtering for a high-confidence difference of at least 10% (right)**.

To detect somatic variants we extend the method of detecting variants by obtaining a posterior probability for each possible difference of frequencies. Figure 5 (left) shows the aggregated posterior of the allele frequency differences for locations not included in the NCBI dbSNP 130 database and restricted further to variant alleles resulting in a non-synonymous change in amino acid. Those criteria were chosen because the goal of the sequencing experiments was to detect nucleotides changes relevant to the oncogenic process. The figure has a spike at difference 0%, which is expected since the majority of mutant alleles are germline variants present in both samples at equal allele frequency. The figure also contains smaller features at ±50% and ±100% due to potential candidates for somatic mutations.

Similar to variant detection, the identification of somatic alleles is based on choosing a suitable cutoff on frequency difference. In the variant detection context our search for suitable frequency cutoff was guided by the frequencies of variants at known (as recorded in NCBI dbSNP 130) locations observed in both tumor and normal samples. To establish a variant difference cutoff we leveraged the validation results of 121 potential somatic alleles 67 of which were confirmed to be genuine somatic mutations present only in the tumor samples.

The list of potential somatic alleles was chosen in the following way. First, starting from a uniform prior on frequencies 0%, 50%, and 100%, with the iterative procedure outlined in the Methods section we constructed an empirical prior for those frequencies. Next, amongst non-synonymous variants whose locations do not appear in dbSNP we selected those with posterior confidence of having zero frequency in tumor at most 10^-6 ^and a posterior confidence of having non-zero frequency in normal at most 10^-6^. Finally, we restricted our attention to the variants having an observed frequency at least 25% in the tumor and observed frequency at most 5% in the normal. Those 121 variants were subjected to validation by a direct Sanger sequencing, and 67 were confirmed to be present in the tumor, but absent from the normal samples.

**Figure 6:**
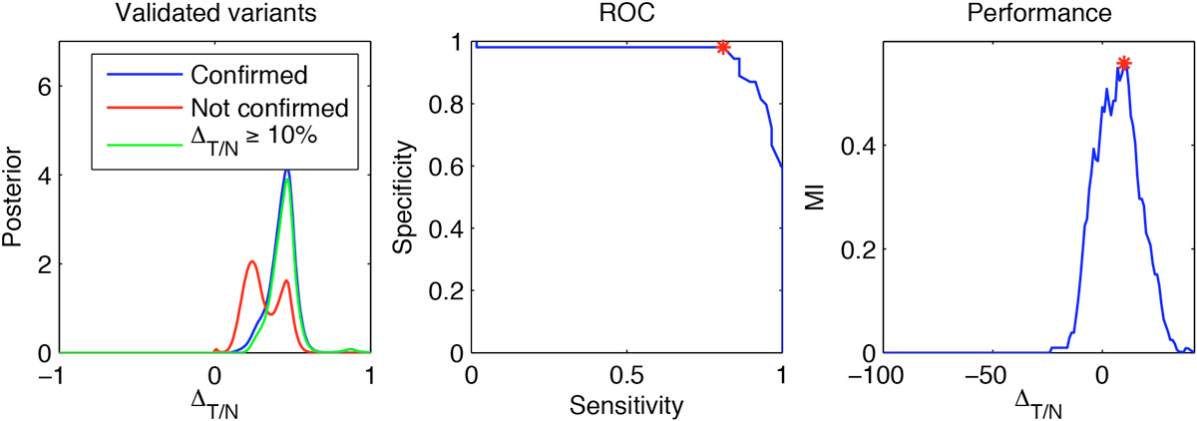
**Establishing frequency cutoff using validated variants**. Confirmed variants have higher frequency difference (left). ROC curve for a varying frequency difference cutoff (center). Obtaining a difference frequency cutoff by maximizing mutual information (right).

Figure 6 (left) shows the aggregated posterior distribution of frequency differences for the validated variants. The variants, which were not confirmed, exhibited a bimodal posterior distribution of frequency differences. This feature was a consequence of the bimodal posterior distribution of the tumor frequency distribution for those variants due to the particular data (quality, variant and total depth) for those variants. Informally, the data for those variants was not sufficient to distinguish a posteriori between the variant being absent or present. In retrospect and having the validation results in which such variants were not confirmed in the tumor, the ability of the posterior to mark them as less reliable was remarkable.

To select a frequency difference able to distinguish between the confirmed variants from those which were not confirmed in the validation experiment, we performed a mutual information analysis, similar to the one we used to obtain a variant frequency cutoff in the context of detection of variants. More precisely, for every possible frequency difference cutoff we compared the variants obtained at this cutoff with the result of the validation experiment and chose the cutoff for which the mutual information of the corresponding contingency table was maximized. In this way, a cutoff of 10% on frequency difference was chosen. Figure 6 (center, right) contains the details of this analysis and Figure 6 (left, in green) contains the aggregated posterior of the validated variants, showing an increase in frequency of at least 10% with high posterior confidence. As designed, this cutoff was able to distinguish the confirmed from the non-confirmed variants quite well, so we went back and filtered the novel non-synonymous variants using this criterion. In addition, we filtered for variants for which a decrease of at least 10% in allele frequency was observed. Our hypothesis was that such variants might be due to a loss of heterozygosity, which was confirmed subsequently. The aggregated posterior distribution of the frequency differences of the filtered variants is given in Figure 5 (right).

## Conclusions

We have presented an empirical Bayesian framework for estimating allele frequencies in a given sample and for comparing such frequencies across samples. The method constructs an empirical prior through an iterative procedure with a fixed-point distribution predicting the empirically observed distribution on data as a marginal. Given the prior, the method combines the data of a particular position with Binomial likelihood to produce a posterior allele frequency distribution. We use high credibility intervals derived from that distribution to decide between the competing variant present/absent hypotheses and distinguish alleles existing in the sample from variants introduced as artifacts by the sequencing technology. For a particular sample involving the study of a human/cancer genome, the parameters of that decision can be inferred from the variants present at locations polymorphic in the human population. Finally, when confronted with a pair of samples, we obtain a posterior distribution on the allele frequency differences between them and detect the alleles presenting a substantial differential. This differential can constitute the appearance of oncogenic alleles, as well as a loss of alleles, e.g. loss of heterozygosity, as a result of the oncogenic process. In our work we used a set of validated variants to deduce the cutoff on frequency difference and have shown that the chosen cutoff distinguishes variants confirmed to distinguish the samples, e.g. somatic variants, from those which do not, e.g. germline variants. Regarding cutoffs we can make the general comment that although obtaining theoretical guarantees on those cutoffs is important, in experiments in which subsequent independent validation is available the stress is more on the cutoff parameter as setting a priority in the validation and leaving the decision of a cutoff to be guided by the design and the resources of the experiment.

This method was developed in order to analyze the sequencing data from tumor and paired normal samples from HCL patients and was successful in identifying important somatic mutations present in the tumor sample. The goal of the project is to obtain the mutational landscape of that disease and will be reported in a separate publication. We saw the study as an opportunity to explore the larger goal of estimating the frequency of alleles in a population of cells. Our motivation for this was enabled by the ability of the novel high-throughput sequencing technologies to detect alleles at a growing depth, and hence at a decreasing frequency. The importance of this to the future of biomedical research is immense, since it will let us fine-tune to a higher precision our understanding of complex populations of cells. More precisely, it will let us detect low frequency alleles, which might have been or will be selected for a clonal expansion in the evolution of that population. Furthermore, as is often the need in biomedical research, this approach will let us study in finer detail the differences between two samples as captured by their genetic makeup. This is important in contexts in which impurities in the sample might confound a more coarsely discretized view of the data. Our conclusion is that the approach to analyzing sequencing data through estimating allele frequencies using empirical Bayes methods is a powerful complement to the ever-increasing throughput of the sequencing technologies.

## Methods

### Bayesian framework

Consider a random source, which produces bits ("0"s and "1"s) with probability/frequency of "1" equal to *f*. Assume also a probability *e *of error in reading the bit. Then the probability of observing a "1" is

$$\left(1-e\right)f+e\left(1-f\right)=f+e-2ef$$

Assume a prior density *p(f) *of the frequency *f *and a prior for the error *e *uniform on the interval [0, *E*] for a fixed 0 ≤ *E *≤ 1. A random experiment produces a string of *m *bits with *n *"1"s. Assuming a binomial likelihood of the data, the posterior density on *(f, e) *is

$$P\left(f,e\right)=\frac{p\left(f\right)}{E\cdot C}{\left(f+e-2ef\right)}^{n}{\left(1-f-e+2ef\right)}^{m-n}$$

where the constant *C *ensures that $\int P\left(f,e\right)dfde=1$. For the marginal posterior on *f *we obtain

$$\begin{array}{llll} P\left(f\right) & =\frac{p\left(f\right)}{E\cdot C}\overset{E}{\underset{0}{\int }}{\left(f+e-2ef\right)}^{n}{\left(1-f-e+2ef\right)}^{m-n}de\; & & \;\\ & =\;\;\frac{P\left(f\right)}{C}B_{n,m}\left(f,f+E-2Ef\right)\; & & \;\\ & \; & \end{array}$$

where

$$B_{m,n}\left(a,b\right)=\frac{1}{b-a}\overset{b}{\underset{a}{\int }}x^{n}{\left(1-x\right)}^{m-n}dx$$

An event/hypothesis *H *is a set of frequencies. We compute the prior *p(H) *and posterior *P(H) *of *H*, by summing/integrating over the frequencies in *H*. Following the Bayesian framework, *p(H) *reflects our prior knowledge about the hypothesis *H *and *P(H) *our belief in the hypothesis after we have observed the data/evidence.

If {*H_1_, ..., H_k_*} is a disjoint and complete set of hypotheses, then $\sum_{i}P\left(H_{i}\right)=1$ and we assume the hypothesis with the highest posterior to hold. Alternatively, given a confidence threshold α, we can find a set of hypotheses, which are satisfied with confidence at least 1 - α. A particular choice for this set orders the hypotheses in decreasing order of their posterior and then selects the smallest *l *so that the total posterior weight of the top *l *hypotheses is at least 1 - α.

A particular example of the above framework is obtained by taking the prior to be concentrated on 0, ½, and 1 with densities *p_0_*, *p_1/2_*, and *p_1_*, i.e.

$$p\left(f\right)=p_{0}\cdot \delta \left(f\right)+p_{1/2}\cdot \delta \left(f-1/2\right)+p_{1}\cdot \delta \left(f-1\right)$$

Consider the hypothesis *H_fair _= {½} *and *H_fake _= {0,1}*. Interpreting the random experiment as a sequence of *m *coin flips of which *n *have come up "Heads", the posterior *P(H_fair_) *reflects our belief that the coin we are observing is fair.

In general, a random experiment can consist of *k *independent pieces of data/evidence *(n_1_, m_1_), ..., (n_k_, m_k_)*. A hypothesis *H *in this setting is a set of sequences of frequencies. For example, for a given frequency *f *the hypothesis *H_f _= {f = f_1 _= ... = f_k_} *consists of all sequences which have all of their components equal to *f*. Since hypotheses are sets, we can combine them with the usual operations permitted on sets, i.e. union, set, and intersection. Thus, to continue our example, we can form the hypothesis *H_eq _*consisting of all sequences with all components being equal, regardless of their common value, as the union $\cup_{f}H_{f}$. We define the prior probability *p(f_1_, ..., f_k_) *of a sequence of frequencies *(f_1_, ..., f_k_) *to be the product *p(f_1_)...p(f_k_) *of the priors of its components. Since the pieces of evidence are independent, we obtain the posterior probability *P(f_1_, ..., f_k_) *of the sequence to be the product *P(f_1_)...P(f_k_) *of the posteriors of its components. The prior/posterior of a hypothesis *H *is formed by summing the priors/posteriors of its elements.

### Genotyping a diploid organism

In sequencing data, each position observed by the sequencing instrument acquires an independent piece of evidence regarding the frequency of the allele at that position. Diploidity means that for each position there are three possible zigosity types: both homozygous and equal to the reference genome (two alleles equal to the reference), heterozygous (exactly one allele equal to the reference), or both homozygous and different from the reference genome (two alleles different from the reference). The reference genome in this case refers to the particular genome to which the reads of the sequencing experiment have been aligned. These three possibilities correspond naturally to three possible frequencies 0, ½, and 1 for the random experiment discussed in the general section on the Bayesian framework, where the frequency of a bit being "1" captures the frequency of the non-reference/variant allele.

In a given sample, the data at a particular position consists of the total number *m *of reads mapping at that position and the number *n *of those reads confirming the variant allele. We interpret the data *(n, m) *as the evidence for the zygosity type of the particular position. Following the Bayesian framework, we compute the posteriors *P_0_*, *P_½_*, and *P_1 _*for the three possible zygosity types and choose the type with highest posterior to be the type of the position. The upper bound *E *on the error in reading the nucleotide allele corresponds to the probability of a non-systematic/random sequencing error. Letting *Q *to be the average of the Phred scores provided by the sequencing instrument for all variant nucleotides mapping at the position, we set *E *to be *10^-Q/10^*.

### Detection of variants in diploid genomes

To detect the presence of a variant at a particular position, we form two hypotheses one for the position being a variant (heterozygous or homozygous) and one for the position being a non-variant (homozygous for the reference), and consider their corresponding posteriors *P_var _*and *P_ref_*.

### Comparing variants across samples from diploid organisms

The discussion so far has focused on the reads obtained from the sequencing of a single sample. In the cancer sequencing experiment discussed in this paper, we have two types of samples for each patient: one from the cancer tissue and one from a normal tissue. The goal is to set the variants obtained from the cancer tissue against the background provided by the normal and obtain the somatic variants which are present in tumor tissue, but do not appear in the normal. Thus, the evidence for the lineage type, i.e. germline vs. somatic, of the variant at a particular position in the genome consists of the two independent pieces of evidence for the zygosity type of that position in the tumor and the normal tissue. Since the data for the zygosity type is simply the total counts, then the evidence for the lineage type is *(n_t_, m_t_) *and *(n_n_, m_n_)*. Using this data one can assign the posterior probability to the hypothesis that the variant is somatic to be

$$P_{som}=P_{t,var}\times P_{n,ref}+P_{t,ref}\times P_{n,var}$$

and then set the non-somatic posterior to *P_nsom _= *1 *- P_som_*.

### Detection of variants in non-diploid genomes

An objection to the approach outlined so far is that although the cancer genome descends from the diploid human genome, the zigosity of a particular allele can be distorted considerably by the oncogenic process, to the point where the diplodity of that allele is dubious. For example, it is known that wildly varying copy numbers are an important characteristic of many cancer genomes. Furthermore, impure samples containing several types of genomes, e.g. mixture of tumor and normal cells, can produce non-diploid frequencies. To account for this, one has to abandon the notion that alleles come in only three possibly frequencies, and allow for more possibilities in the priors and posteriors. Further applications of sequencing experiments, e.g. sequencing cellular transcriptomes or experiments characterizing metagenomic samples, point to the necessity of a wider vocabulary of allele frequencies.

To simplify our exposition, and since the resolution of current sequencing technologies is rather limited, we decided to consider as a possibility the set {0, 0.01, 0.02, 0.03, ..., 1} of frequencies with resolution 0.01. Under this setting for a given position in the sequenced genome we have a hypothesis for every possible frequency *f *of the non-reference allele with is corresponding posterior *P_f _*obtained from the data *(n, m) *for that position. Given the posteriors probability of every possible frequency, one can find the most likely and the expected frequency. Furthermore, given a confidence threshold α, we can obtain a credible interval such that the posterior weight of the frequencies outside of this interval is at most α.

The decision whether a variant allele is present is based on selecting a frequency cutoff *f *and confidence threshold α < 0.5 and then calling present the variants for which the event "frequency at least *f*" has posterior confidence at least 1 - α. The frequency cutoff *f *can be chosen to be equal to 1%, i.e. variant is present, if it has non-zero frequency, or selected according to a set of validated variants, for example from a paired SNP array analysis.

### Comparing variants across samples from non-diploid genomes

The setting can be extended to the comparison of two samples in the following way. For every two frequencies *f_1 _*and *f_2 _*we can obtain their posteriors $P_{1,f_{1}}$ and $P_{2,f_{2}}$ according to each of the samples. Then assuming that the samples are independent we can assign the posterior of the pair $P_{f_{1},f_{2}}$ to be $P_{1,f_{1}}\times P_{2,f_{2}}$. Next, for every difference *Δ *we can form the posterior probability $P_{\Delta }$ that the difference *f_1 _*- *f_2 _*is equal to *Δ*, namely

$$P_{\Delta }=\underset{f_{1}-f_{2}=\Delta }{\sum }P_{f_{1},f_{2}}$$

Finally, we can obtain the most likely and the expected frequency difference, as well as a credible interval for confidence level 1 - α. One can use those posterior measurements to focus on a subset of the alleles, e.g. somatic alleles, which differentiate the two samples.

Similar to the case of detection of variants, the decision whether a variant allele has different frequency in the two samples is based on selecting a frequency difference cutoff *d *and confidence threshold α < 0.5. If one is interested only in variants in which the allele frequency has increased, for example somatic point mutations, then one can focus only on those variants for which the event "frequency difference at least *d*" has posterior confidence at least 1 - α. If in addition the goal is to obtain locations with a loss of allele, e.g. as part of obtaining the loci of a loss of heterozygosity, then the event is "frequency difference at least *d *in absolute value". The frequency cutoff *d *can be chosen to be equal to 1% or adjusted to an existing set of validated variant positions.

### Construction of priors

The prior distribution is an essential component of the Bayesian framework. Generally speaking, the prior captures our belief in a particular hypothesis before we have observed the data. From a practical point of view, when many repetitions of a random experiment are available our belief should coincide with the frequency of occurrence of the hypothesis.

For the application of this framework to sequencing data of diploid organisms, a prior should capture our knowledge about the relative prevalence of the three zygosity types in the genome of the organism. Ignoring our knowledge in that respect, we can set the prior distribution to be uniform over the three types. Concerning a sequencing experiment focused on the coding part of the human genome, a better-informed prior takes into consideration that this part of the human contains around 10^7 ^nucleotides, and that the average number of variants (heterozygous and homozygous and different from the reference) with respect to a reference genome is around 10^4 ^[21]. Furthermore, considering that the reference genome is given as haploid, whereas the data is obtained from the diploid cancer/normal samples one can show that the number of heterozygous variants is expected to be twice as many as the homozygous ones. This holds because fixing the order of the two homologues of the human genome, a heterozygous variant can be located on either one, whereas for the homozygous variant there is a single choice. This leads to a prior in which the proportion of the three zygosity types is roughly 10^3^: 2: 1.

An empirical prior can be obtained if we acknowledge that we have two samples from a patient - from tumor and normal tissue. Hence one can estimate the proportion of the three zygosity types from one of the samples and use this estimate to form the prior for the other. In addition to this, an empirical prior can be obtained form previous sequencing experiment using the same sequencing technology. This approach is applicable also to the more general frequency setting.

More precisely, to obtain an empirical prior we use that a sequencing experiment consists of multiple random experiments - one for each location of the genome. Thus an empirical prior based on a particular sequencing experiment should reflect the allele distribution observed in the genome by that experiment. Using that in the Bayesian framework the prior and the likelihood determine the distribution of the data as a certain marginal, the empirical prior derived from a particular experiment should predict the distribution of the data as observed in that experiment. In particular we must have $p_{e}\left(f\right)=\int P_{e}\left(f|D\right)P_{e}\left(D\right)dD$ where $p_{e}\left(f\right)$ is the empirical prior, $P_{e}\left(f|D\right)$ the posterior based on that prior, and $P_{e}\left(D\right)$ is the empirically observed distribution of the data. Thus we establish the following iterative procedure for constructing an empirical prior. Let $p_{0}$ be the prior before the experiment. In the case of lack of prior knowledge we can set $p_{0}$ to be the uniform distribution. Then on iteration i=0,1,… we set

$$p_{i+1}\left(f\right)=\int P_{i}\left(f|D\right)P_{e}\left(D\right)dD=\frac{1}{N}\overset{N}{\underset{j=1}{\sum }}P_{i}\left(f|D_{j}\right)$$

where summation is over all pieces of data and $P_{i}\left(f|D_{j}\right)$ is the posterior probability of frequency *f *based on prior $p_{i}$ for the *j-*th piece of data. The empirical prior $p_{e}$ is a stationary point of this process. In practice we terminate the process as soon as there is no substantial change in the prior. For the data obtained from the sequencing the human exome and using frequencies of resolution 1% we used around 10 iterations at which point the Kullback-Leibler divergence between the consecutive priors was 0.0013 bits (see Figure 2).

In the case of the human genome the empirical priors obtained in this way were similar to the theoretical prior outlined above and exhibited the expected modes at 0.5 and 1 (variant alleles), and 0 (reference alleles). Furthermore, the frequency distribution in the vicinity of the 0.5 mode was observed to be fitted well by a beta distribution. This empirical observation is consistent with the classically established prescription for choosing the beta distribution as a conjugate prior to a binomial likelihood. Furthermore, the beta distribution was justified because we observed that the depths follow a negative binomial distribution, and hence the observed allele frequencies are a ratio of such distributions, which is approximated well by a beta distribution. As far as the observed negative binomial distribution of the depths, our conjecture is that this is related to the fact that locally the depth distribution is Poisson, but that the means of these Poissons vary widely due to the heterogeneity in the nucleotide content of the human genome and are long tailed over the whole genome. Hence globally the depth distribution is a convolution of Poisson distributions with a long tailed distribution of the means, which is the nature of the negative binomial distribution. The variance of the beta distribution fitting the distribution of the heterozygous alleles depends on the ability of the sequencing technology to detect the two alleles at a heterozygous locus: the more uncorrelated the observed frequencies of the two alleles the wider the variance.

### Choosing confidence cutoffs

Classically, the Bayesian framework prescribes the choice between two competing hypotheses to the one with higher posterior weight. In our case we follow this prescription with the addition that we do not make a decision in the case that the posterior weight of the more likely hypothesis is not high enough. Our decision is justified by the observation that, assuming independent random experiments, maximizing the posterior when deciding on a hypothesis in each will maximize the posterior over all choices because in this case the posterior over all choices is equal to the product of the individual choices. This is not necessarily true if the data is not independent and in this case a more sophisticated analysis is necessary. Furthermore, restricting our attention only to the experiments for which the most likely hypothesis has a high enough posterior guarantees a high enough overall posterior. We answer the question of how high is high enough by noting that if for each experiment the most likely hypothesis has a posterior confidence at least 1 - *α *and we have *N *experiments, then the overall posterior is at least ${\left(1-\alpha \right)}^{N}\approx 1-N\alpha$. Thus to reach a high enough overall posterior confidence, a reasonable posterior confidence for each experiment can be obtained by taking *α *proportional to the inverse of the number of experiments in a manner similar to the Bonferroni correction in the frequentist setting.

Since for current estimates the human exome contains around 10^7 ^nucleotides, in the case of sequencing experiments related to that part of the human genome, when deciding a posteriori between two competing hypothesis, e.g. present/absent or germline/somatic, we can to take *α *= 10^-6 ^for each genomic location.

## Competing interests

The authors declare that they have no competing interests.

## Authors' contributions

VT and RR designed the experiment, developed the method, analyzed the experimental data, and wrote the manuscript. LL designed the experiment and participated in the development of the method. BF and ET conceived the experimental study, designed the experiment, and provided samples for it. All authors read and approved the manuscript.

## Declarations

The publication of this article was funded by the Northeast Biodefence Center (U54-AI057158), the National Institute of Health (U54 CA121852-05), and the National Library of Medicine (1R01LM010140-01).

This article has been published as part of *BMC Systems Biology *Volume 7 Supplement 2, 2013: Selected articles from The 6th International Conference of Computational Biology. The full contents of the supplement are available online at http://www.biomedcentral.com/bmcsystbiol/supplements/7/S2.
